# Global health trials methodological research agenda: results from a priority setting exercise

**DOI:** 10.1186/s13063-018-2440-y

**Published:** 2018-02-05

**Authors:** Anna Rosala-Hallas, Aneel Bhangu, Jane Blazeby, Louise Bowman, Mike Clarke, Trudie Lang, Mona Nasser, Nandi Siegfried, Karla Soares-Weiser, Matt R. Sydes, Duolao Wang, Junhua Zhang, Paula R. Williamson

**Affiliations:** 1 Clinical Trials Research Centre, Biostatistics, University of Liverpool, Liverpool, UK; 2 Academic Department of Surgery, Queen Elizabeth Hospital, Birmingham, UK; 3 Medical Research Council ConDuCT II Hub for Trials Methodology Research, School of Social and Community Medicine, University of Bristol, Bristol, UK; 4 Clinical Trial Service Unit, Nuffield Department of Population Health, University of Oxford, Oxford, UK; 5 Northern Ireland Network for Trials Methodology Research, Queen’s University Belfast, Belfast, UK; 6 The Global Health Network, Centre for Tropical Medicine and Global Health, University of Oxford, Oxford, UK; 7 Peninsula Dental School, Plymouth University, Plymouth, UK; 8 South African Medical Research Council, Cape Town, South Africa; 9 Cochrane, London, UK; 10 Medical Research Council Clinical Trials Unit at University College London, London, UK; 11 Liverpool School of Tropical Medicine, Liverpool, UK; 12 Evidence Based Medicine Centre, Tianjin University of Traditional Chinese Medicine, Tianjin, China; 13 North West Hub for Trials Methodology Research/Clinical Trials Research Centre, University of Liverpool, Liverpool, UK

**Keywords:** Trials methodology, Priority setting, Global health

## Abstract

**Background:**

Methodological research into the design, conduct, analysis and reporting of trials is essential to optimise the process. UK specialists in the field have established a set of top priorities in aid of this research. These priorities, however, may not be reflected in the needs of similar research in low- to middle-income countries (LMICs) with different healthcare provision, resources and research infrastructure. The aim of the study was to identify the top priorities for methodological research in LMICs to inform further research and ultimately to improve clinical trials in these regions.

**Methods:**

An online, two-round survey was conducted from December 2016 to April 2017 amongst researchers and methodologists working on trials in LMICs. The first round required participants to suggest between three and six topics which they felt were priorities for trial methodological research in LMICs. The second round invited participants to grade the importance of a compulsory list of topics suggested by four or more individuals, and an optional list of the remaining topics.

**Findings:**

Rounds 1 and 2 were completed by 412 and 314 participants, respectively. A wide spread of years of experience, discipline, current country of residence, origin of trials training and area of involvement in trials was reported. The topics deemed most important for methodological research were: choosing appropriate outcomes to measure and training of research staff.

**Conclusion:**

By presenting these top priorities we have the foundations of a global health trials methodological research agenda which we hope will foster future research in specific areas in order to increase and improve trials in LMICs.

**Electronic supplementary material:**

The online version of this article (10.1186/s13063-018-2440-y) contains supplementary material, which is available to authorized users.

## Background

Clinical trials are widely recognised as the ‘gold standard’ for estimating treatment effects [1]; however, they are often costly and time-consuming [2]. Methodological research into the design, conduct, analysis and reporting of trials aims to optimise trials so as to yield reliable results in a cost- and time-effective manner. In a previous study the most important methodology research topics for trials within the UK were identified, by the key stakeholder group of directors of UK Clinical Research Centre (UKCRC)-registered clinical trials units (CTUs), as methods to boost recruitment, choosing appropriate outcomes to measure and methods to minimise attrition [3]. However, due to economic, political and cultural differences, it cannot be assumed that priorities for trial method research in the UK mirror either those in other high-income countries or, importantly, those in low- to middle-income countries (LMICs).

LMICs are under-represented within trials methodology research. The 2013 World Health Report encouraged LMICs to become the generators and not just recipients of research data in order for relevant research, according to region-specific health needs and priorities, to result in improvements in public health outcomes [4]. There is a need for methodological research in LMICs to ensure that relevant issues are identified and communicated to healthcare workers in these regions so that they might optimise future designs for trials.

The aim of the study was to identify the top priorities for methodological research according to researchers and methodologists working in LMIC trials.

## Methods

A two-round online survey was conducted from December 2016 to April 2017, targeting researchers who had designed, conducted or analysed trials in LMICs. An invitation letter, describing the scope of the study and providing a hyperlink to the survey, was circulated amongst members of the Global Health Network, the European and Developing countries Clinical Trials Partnership (EDCTP) and other networks identified via the authors. The registry ClinicalTrials.gov was used to search for trials currently open to recruitment in LMICs. Researchers involved in these trials who had provided an email address were contacted. Countries were deemed as LMICs according to the Organisation for Economic Co-operation and Development (OECD) Development Assistance Committee (DAC) list of Official Development Assistance (ODA) recipients in 2016 [5].

The survey was conducted in English and translations of the invitation letter in French, Spanish and Chinese were also disseminated in order to help the targeted participants understand the scope of the study. These translations were either back-translated or checked by a second individual to ensure that no information was lost or changed during the translation process.

In round 1, an initial eligibility question identified health professionals and methodologists who had clinical trial experience in LMIC settings. Participants were asked about their professional background, current country of residence and invited to provide between three and six topics that they believed should be priorities for trials methodology research in LMICs. Trials methodology research was defined as research investigating the methods, practices and procedures that are used for the design, conduct, analysis, interpretation and reporting of clinical trials. Topics were categorised and reviewed by the Steering Committee, and those deemed not applicable or beyond the scope of the survey were excluded. A primary list of topics identified by four or more respondents was created in order to decrease participant burden in the second round thereby increasing the likely response rate, and the remaining topics were included in a secondary list. In order to aid comparison between the UK and LMIC priorities, topics in the primary list for the UK survey which were not suggested in the current survey were also added to the secondary list.

The hyperlink to round 2 of the survey was circulated to those who provided an email address in round 1. These first-round participants were sent weekly reminders to complete the survey via email to maximise the response rate. Round-2 participants were again required to provide information about their professional backgrounds and then to assign the topics in each list a score, using the GRADE guidelines scale [6] in order to identify the more important research topics. Scores of 1 to 3 indicated that the topic was not important, 4 to 6 important but not critical, and 7 to 9 critically important. So as to reduce participant burden, assigning of grades to topics in the primary list was compulsory for completion but optional for topics in the secondary list. As an incentive, those who completed both rounds of the survey were included in a prize draw to win travel to, and accommodation at, the joint International Clinical Trials Methodology Conference and Society of Clinical Trials 2017 Conference in Liverpool, UK. Completion of the survey was deemed consent to participate.

## Results

**Fig. 1:**
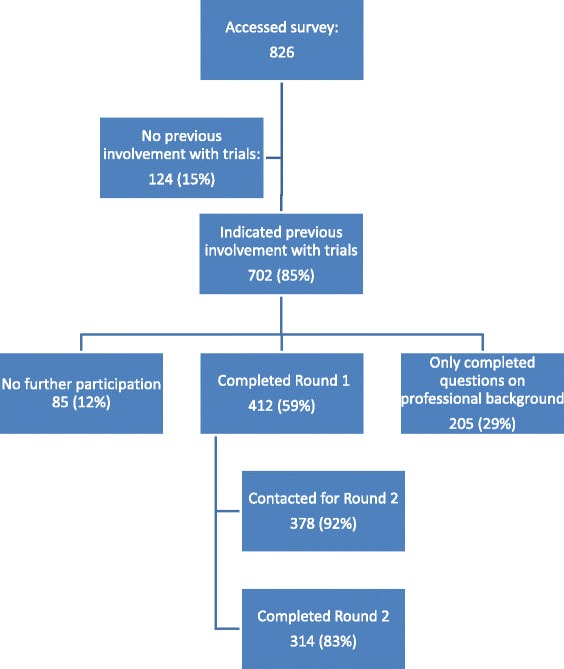
Flow chart showing participation throughout the survey

Round 1 of the survey was accessed by 826 people; 124 (15%) indicated no previous involvement in clinical trials and thus did not continue. Of the remaining participants, 85/702 (12%) participants did not answer any further questions after indicating involvement in trials, 205/702 (29%) completed questions about their professional backgrounds only and 412/702 (59%) completed round 1 by providing at least one priority topic (Fig. 1).

Table 1 shows the demographic and professional characteristics of the completers of round 1 of the survey. The number of years of experience working in clinical trials for those who completed round 1 ranged from 1 to 45 years, with a mean (standard deviation) of 7.7 (7.0) years.

**Table 1:** Professional background and demographic details for survey completers

	Round 1 *N* = 412	Round 2 *N* = 314
Years of experience working in clinical trials		
Mean (SD)	7.67 (7.02)	7.89 (7.14)
Median (IQR)	5 (3, 10)	6 (3, 10)
Range	1, 45	1, 50
Participant disciplines		
Clinical disciplines	268 (65%)	196 (62%)
Public health	59 (14%)	47 (15%)
Infectious diseases	57 (14%)	40 (13%)
General medicine	48 (12%)	27 (9%)
Paediatrics	24 (6%)	14 (4%)
General surgery	17 (4%)	13 (4%)
Nursing	9 (2%)	7 (2%)
Obstetrics	8 (2%)	12 (4%)
Primary care	8 (2%)	7 (2%)
Psychiatry	8 (2%)	3 (1%)
Dentistry	5 (1%)	2 (1%)
Oncology	5 (1%)	4 (1%)
Physiotherapy	3 (1%)	3 (1%)
Cardiology	2 (<1%)	1 (< 1%)
Gynaecology	2 (< 1%)	1 (< 1%)
Haematology	2 (< 1%)	2 (1%)
Neurosurgery	2 (< 1%)	1 (< 1%)
Anaesthesia	1 (< 1%)	1 (< 1%)
Dermatology	1 (< 1%)	1 (< 1%)
Ear, nose and throat diseases	1 (< 1%)	1 (< 1%)
Neurology	1 (< 1%)	0 (0%)
Nutrition	1 (< 1%)	2 (1%)
Orthopaedics and trauma	1 (< 1%)	6 (2%)
Pneumology	1 (< 1%)	0 (0%)
Rheumatology	1 (< 1%)	1 (< 1%)
Urology	1 (< 1%)	0 (0%)
Research methods disciplines	85 (21%)	61 (19%)
Trials management	37 (9%)	19 (6%)
Statistics	19 (5%)	16 (5%)
Data management	13 (3%)	10 (3%)
Epidemiology	7 (2%)	8 (3%)
Ethics	3 (1%)	2 (1%)
Quality assurance	2 (< 1%)	2 (1%)
Clinical research	2 (< 1%)	2 (1%)
Health economics	1 (< 1%)	1 (< 1%)
Information systems	1 (< 1%)	0 (0%)
Mathematics	0 (0%)	1 (< 1%)
Laboratory science disciplines	37 (9%)	44 (14%)
Biomedical sciences	12 (3%)	27 (9%)
Pharmacy	12 (3%)	10 (3%)
Parasitology	4 (1%)	5 (2%)
Immunology	3 (1%)	0 (0%)
Biology	2 (< 1%)	0 (0%)
Microbiology	2 (< 1%)	1 (< 1%)
Biotechnology	1 (< 1%)	1 (< 1%)
Chemistry	1 (< 1%)	0 (0%)
Other disciplines	22 (5%)	13 (4%)
Health management (administration)	6 (1%)	3 (1%)
Social sciences	6 (1%)	4 (1%)
Community engagement	3 (1%)	1 (< 1%)
Complementary medicine	3 (1%)	2 (1%)
Pharmacology	2 (< 1%)	1 (< 1%)
Medical devices	1 (< 1%)	0 (0%)
Pharmacogenomics	1 (< 1%)	0 (0%)
Global health	0 (0%)	1 (< 1%)
Environmental heath	0 (0%)	1 (< 1%)
Origin of trial experience		
In a low- to middle-income country (LMIC) only	250 (61%)	196 (63%)
In both a LMIC and a high-income country (HIC)	100 (24%)	76 (24%)
In a HIC only	62 (15%)	42 (13%)
Involvement in clinical trials		
Design	240 (58%)	174 (55%)
Conduct	361 (88%)	267 (85%)
Analysis	213 (53%)	171 (54%)
Reporting	257 (62%)	200 (64%)
Current residence by continent		
Africa	210 (51%)	171 (54%)
Asia	82 (20%)	55 (18%)
Europe	64 (16%)	41 (13%)
South America	32 (8%)	30 (10%)
North America	23 (6%)	16 (5%)
Australia	1 (< 1%)	1 (< 1%)

*SD* standard deviation, *IQR* interquartile range

Approximately half of the participants (210/412, 51%) reported to be currently residing in a country in Africa and over 80 different countries were represented. A variety of clinical disciplines was represented, the majority being public health (14%), infectious diseases (14%) and general medicine (12%); however, researchers with trial-specific roles (trial management, data management and statistics) also participated.

Over 60% of participants received their clinical trial training in a LMIC only. Most researchers reported involvement in the conduct of trials (88%) with over 50% having experience in each of the design, analysis and reporting stages.

The 205 participants who gave information about their professional backgrounds but did not complete the survey were similar to those who completed the survey (Additional file 1: Table S1).

Those who completed round 1 and provided an email address (378/412, 92%) were contacted to participate in round 2. A total of 314/378 (83%) participated. The characteristics of the subgroup that completed round 2 were similar to those of the whole group (Table 1).

**Fig. 2:**
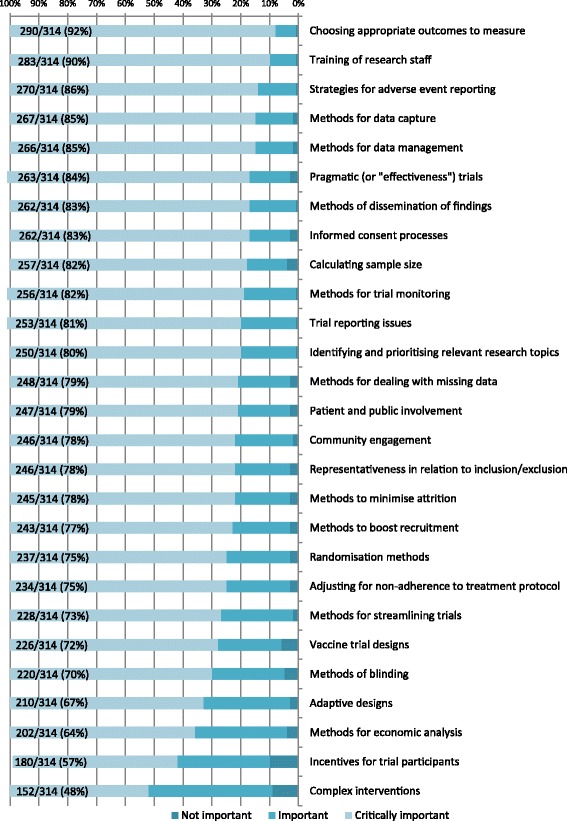
Topics identified by four or more as priorities, ordered by importance ranks

**Fig. 3:**
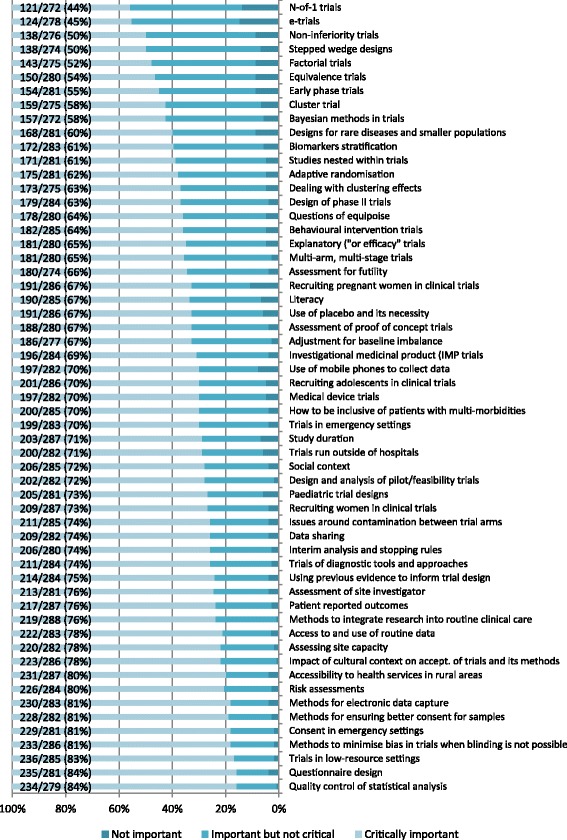
Topics identified by fewer than four as priorities, ordered by importance ranks

A total of 1620 topics were identified, of which 703 (43%) were deemed not applicable due to being too vague or beyond the scope of the study (raw data showing the classifications can be made available on request). The remaining 917 topics were categorised and divided into two lists. The primary list was limited to the 27 topics suggested by four or more participants (Fig. 2). The secondary list comprised the 55 remaining topics suggested and two identified as a priority from the previous UK study but not identified in the current study, giving a total of 57 topics (Fig. 3).

Over 50% of the topics which were scored as critically important by 80% or more responders on both LMIC lists 1 and 2 were around issues in trial conduct, including: training of research staff, strategies for adverse event reporting, methods for data capture, methods for data management, informed consent processes, methods for trial monitoring, consent in emergency settings, methods for ensuring better consent for samples, methods for electronic data capture, risk assessments, accessibility to health services in rural areas.

The top priority for further methodological research for trials in LMICs is appropriate choice of outcomes to measure. Some examples of topics falling within choosing appropriate outcomes to measure, given by respondents, were: developing the correct objectives for the study, standardising outcome sets and identifying patient-focussed endpoints. The second top priority is methods related to training of research staff; for example, finding cost-effective and purposeful methods to train research staff and the use of blended learning incorporating new technologies.

Choice of appropriate outcomes to measure, methods for the conduct of pragmatic trials and calculating sample size all appeared in the top 10 most important items in both the LMIC and UK priority lists [2].

## Discussion

Many of the topics deemed most important to LMIC researchers were related to trial conduct as opposed to trial design or analysis. This could stem from resource issues and may indeed highlight the requirement for capacity development, stressing the need for cost and time-effective methods. The majority (85%) of round-2 participants have been involved in the conduct of trials and, therefore, issues around trial conduct could be more relevant to them.

The priority most commonly graded as critically important, choosing appropriate outcomes to measure, was also a priority identified in the UK study and there is ongoing work on this through the Core Outcome Measures in Effectiveness Trials (COMET) Initiative [7]. Launched in January 2010, COMET aims to optimise the choice of outcomes by providing a standardised set of outcomes (core outcome sets) for specific disease areas and/or populations. Up until December 2015 only 44/248 (18%) completed core outcome set studies involved participants from LMICs. Given that choosing appropriate outcomes to measure was the topic most important to researchers in LMICs, there is a need for greater involvement of these countries in the development of core outcome sets.

It is also important to LMIC researchers to prioritise research into methods for training research staff. One example of implementing and reviewing a training programme is within the Good Health Research Practice (GHRP) initiative which aims to train researchers in applying Good Clinical Practice (GCP). A short course using an experimental learning cycle, the process of conceptualising, applying, acting and reflecting, was piloted between 2014 and 2015 in LMICs [8]. New methods to improve the training programme were identified during the pilot phase and incorporated into future programmes. Research should now be done to find methods for training which are available and effective in LMICs.

The results suggest that, although some research priorities seem to be applicable to both LMICs and high-income countries, differences may exist between these broad regions. For example, in the UK there was a greater emphasis on recruitment and retention, yet these topics did not appear in the top 10 most commonly graded as critically important by LMIC researchers, potentially due to the fact that involvement in trials guarantees access to more personalised healthcare which, outside of a trial setting, could be limited in LMICs due to capacity issues or the intervention not being available outside the trial [9, 10].

Although snowball sampling methods were used to disseminate the survey, which sometimes raises concerns with respect to representativeness, information on demographic details and professional backgrounds of the participants indicated that a wide spread of disciplines and countries were involved, thus strengthening the applicability of the results.

One limitation to note was that participants were researchers and, therefore, there was no patient and public involvement (PPI); this was due to pragmatic reasons, since it would be difficult to identify participants from trials in LMICs; however, it would be useful to obtain their views. It is important that further research based on these results includes PPI so as to conduct research into methods which are also relevant and applicable to patients and the public.

Another limitation of the survey was around the number of topics suggested which were deemed not applicable or beyond the survey scope. Those deemed not applicable were often too vague; for example, ‘trial logistics’, ‘statistical analysis’ and ‘improving trial efficiency’ or to do with a specific disease area; for example, ’HIV’, ‘malaria’; a full list of responses and groupings are provided as a Additional file 2. Participants had space to report the reason for suggesting each topic and where uncertainties to do with applicability arose, this information was used to aid decision-making. Some topics deemed not applicable may have been due to language barriers; however, translations of the invitation letters should have minimised this risk. The invitation letter was translated to French and checked by speakers who were fluent, although not native. The Chinese and Spanish versions were both translated and checked by native speakers. The survey, itself, was not translated.

Furthermore, it is possible that the 29% of people who completed background information in round 1 but did not provide any priorities perhaps did not believe there were any priorities for methodological research. However, due to the ‘free-text’ nature of the survey it is assumed that if participants completed background questions, and felt strongly that there were no priorities, a comment would have been left to indicate this.

Although LMICs share the same limitation of resource issues, it should also be noted that the specific needs of different regions within LMICs could vary; for this reason a wide spread of countries was included in the survey. An extension of this work; however, could target the priorities of specific countries or regions within LMICs.

A variety of disciplines was represented in the survey but it could also be the case that priorities vary depending on respondent affiliations (for example, private vs public).

These findings provide a preliminary step towards achieving the foundations of a global health trials methodological agenda which we hope will foster methodology research in specific areas in order to increase and improve trials in LMICs.

## Conclusions

Choosing appropriate outcomes to measure and methods of training research staff were the top priorities for trialists in LMICs.

## Additional files

Additional file 1: Table S1. Professional background and demographic details for round 1 non-completers. (DOC 70 kb)

Additional file 2: Categories for Survey Round 2 v1.0. (XLSX 96 kb)

## Abbreviations

CTU: Clinical trials unit
DAC: Development Assistance Committee
EDCTP: European and Developing countries Clinical Trials Partnership
GCP: Good Clinical Practice
GHRP: Good Health Research Practice
LMIC: Low- to middle-income country
ODA: Official Development Assistance
OECD: Organisation for Economic Co-operation and Development
UKCRC: UK Clinical Research Centre

## References

[1] Pocock SJ, Elbourne DR (2000). Randomized trials or observational tribulations?. N Engl J Med.

[2] Collier R (2009). Rapidly rising clinical trial costs worry researchers. CMAJ.

[3] Tudur Smith C, Hickey H, Clarke M, Blazeby J, Williamson PR (2014). The Trials Methodological Research Agenda: results from a priority setting exercise. Trials.

[4] Dye C, Boerma T, Evans D, Harries A, Lienhardt C, McManus J, et al. World Health Organisation. The World Health Report 2013: Research for universal health coverage. http://www.who.int/whr/2013/report/en/. Accessed 14 July 2017.

[5] OECD DAC List of ODA Recipients. http://www.oecd.org/dac/stats/documentupload/DAC%20List%20of%20ODA%20Recipients%202014%20final.pdf. Accessed 14 July 2017.

[6] GRADE Working Group. http://www.gradeworkinggroup.org/. Accessed 14 July 2017.

[7] Williamson PR, Altman DG, Bagley H, Barnes KL, Blazeby JM, Brookes ST, et al. The COMET Handbook: version 1.0. Trials. 2017;18(Suppl 3):280. 10.1186/s13063-017-1978-4 PMC5499094 28681707

[8] Mahendradhata Y, Nabieva J, Ahmad RA, Henley P, Launois P, Merle C (2016). Promoting good health research practice in low- and middle-income countries. Glob Health Action.

[9] Abrams A, Siegfried N, Geldenhuys H (2011). Adolescent experiences in a vaccine trial: a pilot study. S Afr Med J.

[10] Ramjee G, Coumi N, Dladla-Qwabe N, Ganesh S, Govinden R, Guddera V (2010). Experiences in conducting multiple community-based HIV prevention trials among women in KwaZulu-Natal, South Africa. AIDS Res Ther.

