# Supplementary material for: Global health trials methodological research agenda: results from a priority setting exercise
Source: Trials. 2018 Feb 5;19:48. doi: 10.1186/s13063-018-2440-y (PMC5798175; doi:10.1186/s13063-018-2440-y)
Supplement: Supplementary file 1 — Professional background and demographic details for round 1 non-completers. (DOC 70 kb) [file 13063_2018_2440_MOESM1_ESM.doc]

Table S1: Professional background and demographic details for Round 1 non-completers

| **Years of experience working in trials** | **N=204** |
| --- | --- |
| Mean (SD) | 6.58 (6.44) |
| Median (IQR) | 5 (2, 9.5) |
| Range | 1, 40 |
| Missing | 1 (<1%) |
| **Participant disciplines** | **N=205** |
| **Clinical disciplines** | **127 (62%)** |
| *Public health* | 35 (17%) |
| *Infectious diseases* | 22 (11%) |
| *General medicine* | 17 (8%) |
| *Paediatrics* | 12 (6%) |
| *General surgery* | 11 (5%) |
| *Nursing* | 9 (4%) |
| *Obstetrics* | 7 (3%) |
| *Primary care* | 4 (2%) |
| *Gynaecology* | 3 (1%) |
| *Orthopaedics and trauma* | 2 (1%) |
| *Anaesthesia* | 1 (<1%) |
| *Cardiology* | 1 (<1%) |
| *Haematology* | 1 (<1%) |
| *Oncology* | 1 (<1%) |
| *Rheumatology* | 1 (<1%) |
| **Research methods disciplines** | **39 (19%)** |
| *Trials management* | 22 (11%) |
| *Data management* | 6 (3%) |
| *Statistics* | 6 (3%) |
| *Ethics* | 2 (1%) |
| *Information systems* | 2 (1%) |
| *Epidemiology* | 1 (<1%) |
| **Laboratory science disciplines** | **21 (10%)** |
| *Biomedical sciences* | 8 (4%) |
| *Pharmacy* | 4 (2%) |
| *Biochemistry* | 3 (1%) |
| *Microbiology* | 3 (1%) |
| *Biotechnology* | 2 (1%) |
| *Biology* | 1 (<1%) |
| **Other disciplines** | **18 (9%)** |
| *Social sciences* | 7 (3%) |
| *Psychiatry* | 6 (3%) |
| *Health management (administration)* | 4 (2%) |
| *Pre-hospital immediate care* | 1 (<1%) |
| **Origin of trials experience** | **N=205** |
| In a low to middle income country (LMIC) only | 135 (66%) |
| In both a LMIC and HIC | 40 (20%) |
| In a high income country (HIC) only | 30 (15%) |
| **Involvement in clinical trials** | **N=205** |
| Design | 81 (40%) |
| Conduct | 148 (72%) |
| Analysis | 72 (35%) |
| Reporting | 93 (45%) |
| **Current residence by continent** | **N=205** |
| Africa | 111 (54%) |
| Asia | 33 (16%) |
| Europe | 33 (16%) |
| South America | 14 (7%) |
| North America | 10 (5%) |
| Australia | 4 (2%) |
